# Does Genotype Affect the Efficacy of PCSK9 Inhibitors in the Treatment of Familial Hypercholesterolemia?

**DOI:** 10.1007/s10557-023-07505-5

**Published:** 2023-08-23

**Authors:** Fistra Janrio Tandirerung

**Affiliations:** Institute of Cardiovascular Science, University College London, Gower Street, London, WC1B 6BT UK

**Keywords:** Genotype, Familial hypercholesterolemia, Low-density lipoprotein cholesterol (LDL-C), Proprotein convertase substilin/kexin 9 (PCSK9)

## Abstract

**Purpose of Review:**

This review discusses whether patients’ genotype affects the efficacy of PCSK9 inhibitors in treating familial hypercholesterolemia and how this might influence clinical management.

**Recent Findings:**

Currently, available evidence consistently demonstrates and is in good agreement that, in general, the LDL-C-lowering effect of PCSK9 inhibitors is similar across genotypes, except for compound heterozygous and homozygous familial hypercholesterolemia (FH). However, it remains to be seen whether the comparable therapeutic effect in lowering LDL-C level also leads to a comparable degree of cardiovascular risk reduction with different genotypes.

**Summary:**

Generally, the level of LDL-C reduction following PCSK9 inhibitor treatment is similar within different genotypes. Hence, genotype is a less reliable predictor for further LDL-C level reduction on PCSK9 inhibitor therapy, and attention should be given to other external influences, especially for heterozygous FH.

## Introduction

Familial hypercholesterolemia (FH) is the most common genetic disease affecting lipid metabolism, regardless of ethnicity [1, 2]. In general, the prevalence of FH can be 1 case in every 250 individuals [1]. Despite the discovery of some other genetic mutations [3], the vast majority of FH cases are due to mutations in the low-density lipoprotein receptor (*LDLR*), apolipoprotein-B (*APOB*), or proprotein convertase substilin/kexin 9 (*PCSK9*) genes [4]. These gene mutations are responsible for a significant increase in low-density lipoprotein (LDL) cholesterol (LDL-C) from the early years of life, which subsequently leads to the early onset of coronary heart disease (CHD) [5, 6].

However, the aforementioned gene mutations are not always identified in individuals clinically diagnosed with FH. This leads to another disease entity called polygenic hypercholesterolemia, secondary to multiple LDL-C-raising genetic variants, which can be confirmed using the polygenic risk score (PRS) instrument [5, 7]. A recent review has elaborated that FH and polygenic hypercholesterolemia have a significantly different response to LDL-C-lowering therapy and cardiovascular disease risk profile [8]. Moreover, different gene mutations also respond differently to therapy. Statins were found to be more effective on *APOB* mutations compared to *LDLR* mutations [9]. Different *LDLR* mutations also tend to show a different degree of LDL-C-lowering on statin therapy [10–12]. Generally, *LDLR* mutations are considered to have the worst phenotype and response to therapy relative to *APOB* and *PCSK9* mutations [9]. This evidence indicates the significance of identifying a patient’s genotype in providing precise and comprehensive clinical management in the context of hypercholesterolemia.

However, the necessity for genotype identification and differentiation in FH and polygenic hypercholesterolemia therapy with PCSK9 inhibitors remains unclear. Some evidence, for example, indicated conflicting results with statin therapy as the therapeutic response to PCSK9 inhibitors seemed to be comparable in FH and polygenic hypercholesterolemia [1, 13]. Therefore, the purpose of this review is to discuss whether a patient’s genotype affects PCSK9 inhibitor efficacy in the treatment of familial hypercholesterolemia and how this might influence clinical management.

## Genetics in Familial Hypercholesterolemia

### LDL Receptor (LDLR) Mutation

Approximately 80% of FH with known mutations are caused by *LDLR* mutations [14], with over 2000 rare mutations having been identified [2]. LDLR on target cells (mainly hepatocytes) binds and uptakes LDL-C, thus reducing plasma LDL-C following LDL-C/LDLR complex internalization into cells. Hence, FH secondary to *LDLR* mutations is due to a loss-of-function mutation that occurs through several mechanisms [15]. These include single nucleotide variants (SNVs) that cover missense mutations (cause amino acid changes) in up to 50% of cases, insertions or deletions (up to 20%) that might change the reading frame, nonsense mutations (around 15%) leading to stop codons, and splicing mutations (around 10%) which occur at intron–exon splicing sites that impair mature mRNA formation. Furthermore, copy number variants (CNV) such as deletion, insertion, or duplication, leading to large gene rearrangements, can also be the underlying mechanism [2, 15]. Some examples of identified *LDLR* mutations are Schlüter et al., who found a deletion of 86 base pairs in exon 5 in first-degree-related patients [16], and Flores et al., who identified a missense mutation (Asp360His) in a Mexican community [17].

The *LDLR* gene consists of 860 amino acids encoded by 18 exons that make up different domains (distinct structural and/or functional protein units regulating a particular function) in the LDLR protein. Mutations can be dispersed in different domains, resulting in different functional impairments [9]. The mutation effect can be an impaired LDLR synthesis (null mutation) or defective mutations ranging from inability to migrate from the endoplasmic reticulum upon synthesis, impaired binding with apolipoprotein-B (Apo-B), internalization impairment after Apo-B binding, or defect in recycling LDLR [2, 9, 10]. Null mutations can be predicted to have a higher LDL-C level compared to defective mutations. This corresponds to the LDLR functional reserve, as the null allele has < 2% of normal LDLR function, while defective mutations range from 2 to 70% [3]. *LDLR* mutations also have a gene-dosage effect. Hence, single heterozygous mutations (a mutation affecting one allele) tend to have lower LDL-C compared to compound heterozygous (each allele has a different mutation), while compound heterozygous is lower than homozygous (the same mutation affecting both alleles) mutations [4].

The magnitude of the mutation effect is in line with the degree of gene rearrangement. Large structural rearrangements secondary to CNVs, splicing variants affecting promoter regions (starting point of gene transcription), and nonsense mutations that prematurely terminate protein synthesis resulting in a shorter amino acid sequence can lead to a null mutation and higher LDL-C, while missense mutations tend to show a lower LDL-C level as they change only a single amino acid [2, 14, 15].

### Apolipoprotein-B (APOB) Mutation

Five percent of FH cases are caused by the *APOB* gene mutation [14]. *APOB* in LDL molecules functions as a ligand that facilitates LDL-LDLR binding on the target cells (mainly hepatocytes) and maintains LDL structural integrity [2, 6, 15]. Hence, *APOB* loss-of-function mutations impair the LDL-LDLR binding process, leading to defective LDL-C plasma clearance [15]. Several mutations have been identified in the *APOB* gene. However, the p.Arg3527Gln mutation is the most commonly found *APOB* mutation (2–5% of FH in the European population) [9, 15, 18]. A different missense mutation at the same location (Arg3527Trp) is predominant in the Chinese population [9].

### Proprotein Convertase Subtilisin/Kexin Type 9 (PCSK9) Mutation

*PCSK9* mutations account for < 1% of FH with known mutations [2]. Once the LDL-C/LDLR complex is internalized, LDL-C will be degraded into cholesterol and amino acids, while LDLR is recycled for another LDL-C uptake cycle on the cell surface [19]. However, when PCSK9 binds to LDLR in the extracellular space, the LDLR/PCSK9 complex will be degraded in the lysosome after internalization (LDLR is not recycled) [20]. Hence, FH from *PCSK9* mutations is the result of a gain-of-function mutation that reduces LDLR availability [2, 21].

Considerable evidence demonstrates that missense mutations are responsible for the *PCSK9* gain-of-function mutation. For example, in 2003, Abifadel et al. found two missense mutations in the *PCSK9* gene (S127R and F216L) [22]. Later in 2010, the *PCSK9* E32K mutation was confirmed to be another FH-causing mutation in the Japanese population [23]. The Asp374Tyr mutation is associated with premature CHD and is common in Norway and the UK [21, 24].

For clarity, a summary of different patient genotypes and terminology is provided in Table 1.

**Table 1:** Definition of genetic terminology in familial hypercholesterolemia [3, 4, 8, 9]

Genotype	Definition
Monogenic familial hypercholesterolemia (FH)	Hypercholesterolemia due to mutations in a candidate gene known to cause FH (mainly *LDLR*, *APOB*, and *PCSK9*)
Polygenic hypercholesterolemia	Hypercholesterolemia due to LDL-C-raising variants and not caused by a single gene mutation known to cause FH
Homozygous FH (HoFH)	FH due to the same mutation on both alleles in one of the known causative genes (bialleleic mutation)
Heterozygous FH (HeFH)	FH due to a mutation in one allele only; the other allele is unaffected
Compound heterozygous FH	Two different mutations in the same or different genes are known to cause FH
Null *LDLR* mutation	*LDLR* mutation leading to < 2% LDLR normal function left
Defective *LDLR* mutation	*LDLR* mutation leading to 2–70% LDLR normal function left
HoFH *LDLR* null (*LDLR* null/null HoFH)	Null *LDLR* mutation in which the same mutation affecting both alleles
HoFH *LDLR* defective	Defective *LDLR* mutation where the same mutation affecting both alleles
HeFH *LDLR* null	Null *LDLR* mutation affecting a single allele in one of the genes known to cause FH
HeFH *LDLR* defective	Defective *LDLR* mutation affecting a single allele in one of the genes known to cause FH
Homozygous *APOB*	The same mutation affecting both alleles of *APOB* gene
Heterozygous *APOB*	An *APOB* gene mutation in one allele
Homozygous *PCSK9*	Both alleles of PCSK9 gene are impaired with the same mutation
Heterozygous *PCSK9*	A mutation impairs an allele of PCSK9 gene

## PCSK9 Inhibitor Overview

Abifadel et al. first discovered in 2003 that gain-of-function mutations in the *PCSK9* gene were responsible for FH [22]. This further provided a rationale for the development of PCSK9 inhibitors (PCSK9i), which, as indicated by the name, inhibit PCSK9 that promotes LDLR degradation. Increased LDLR degradation reduces LDL-C cellular uptake from circulation, leaving LDL-C high in plasma. Thus, the inhibition of PCSK9 with PCSK9i preserves LDLR function, leading to preserved or increased LDL-C plasma uptake into cells, thereby reducing the amount of LDL-C in the circulation [25].

Currently, there are several available PCSK9 inhibition approaches. The earliest PCSK9 inhibitors are in the form of monoclonal antibodies (mAb). PCSK9i monoclonal antibodies competitively bind with PCSK9, which eventually prevents the binding of PCSK9 and LDLR on the cell surface, rendering PCSK9 unable to degrade LDLR on the cell surface [26]. There are only 2 monoclonal antibody PCSK9 inhibitors that have been approved for clinical use by the Food and Drug Administration (FDA) and European Medicines Agency (EMA), alirocumab and evolocumab [25]. The ODYSSEY OUTCOME trial on alirocumab showed 54.7–62.7% of LDL-C reductions at 3-time points (4, 12, and 48 months since randomization) compared to placebo in individuals optimally treated with statins. These results were accompanied by a reduction in ischemic cardiovascular events [27]. Similar results were also documented with evolocumab in the FOURIER trial that demonstrated a 59% LDL-C reduction with a significant cardiovascular event reduction after 48 weeks [28]. A significant LDL-C reduction from baseline was also documented with alirocumab in HoFH patients. The ODYSSEY HoFH trial showed a 35.6% mean LDL-C reduction difference between alirocumab and placebo at 12 weeks of therapy (*p* value =  < 0.0001) [29]. In the RUTHERFORD-2 trial carried out on 331 HeFH patients, evolocumab demonstrated 59.2% and 61.3% LDL-C reductions with biweekly and monthly dosing, respectively [30].

Another PCSK9 inhibition approach is through gene silencing using small interfering ribonucleic acid (siRNA), inclisiran. Inclisiran works intracellularly in hepatocytes by binding to the RNA-induced silencing complex (RISC). The inclisiran-RISC complex subsequently cleaves the mRNA that encodes PCSK9. This results in reduced PCSK9 protein synthesis and increased LDLR and LDL-C uptake [31]. The ORION-9, ORION-10, and ORION-11 trials on inclisiran demonstrated approximately 50% LDL-C reduction as compared to placebo [32, 33]. It is also worth mentioning that PCSK9 inhibition can also be done through an antisense oligonucleotide that is complementary to the sense strand of PCSK9 mRNA, which inhibits PCSK9 protein synthesis [19]. This drug has shown a 43% LDL-C reduction in a mouse study [34].

Importantly, as already reviewed elsewhere [35], in the ODYSSEY Outcome study, alirocumab also pronouncedly reduced the risk of major adverse cardiovascular events (MACE) and deaths in patients with concomitant coronary and peripheral artery disease (PAD) involvement [36]. This result is similar to the FOURIER cohort in that the addition of evolocumab to statin in PAD patients significantly reduced MACE and major adverse limb events such as acute limb ischemia, urgent revascularization, and major amputation. The lower the LDL-C level attained, the lower the risk of lower limb events without increasing safety concern [37].

Despite both mAb and siRNA PCSK9 inhibitor LDL-C-lowering efficacy and tolerability tend to be comparable, there are some distinct differences between the 2 groups. Compared to mAb PCSK9 inhibitors, inclisiran has a higher durability, can be administered twice a year, and might reduce PCSK9 level intracellularly and extracellularly [38, 39]. However, inclisran’s effect on reducing cardiovascular events and its long-term safety are yet to be confirmed [40].

However, currently approved PCSK9 inhibitors are administered with injection that may lead to convenience or tolerability concerns. Thus, an orally administered PCSK9 is now under development, that in a phase 2b clinical study, was found to be well-tolerated and capable of reducing up to 60% LDL-C from baseline in a dose-dependent manner. These results, despite excluding HoFH patients, were observed in study participants with varying demographic status, cardiovascular risks, and statin background therapy [41].

## The Impact of Genotype on LDL-C Level Following PCSK9 Inhibitor Therapy

A study done by D’Erasmo et al. [1] studied 370 patients divided into monogenic FH (209), polygenic hypercholesterolemia (89), and genetically undetermined (72). All patients were initially treated with lipid-lowering therapy (LLT) of different intensities, and during follow-up, 37 individuals received PCSK9i (evolocumab or alirocumab). Despite the fact that monogenic FH had the poorest therapeutic response compared to the other groups, the desired LDL-C level was only achieved after PCSK9i was administered. More importantly, the LDL-C-lowering effect of PCSK9i seemed to be independent of genotype. Furthermore, at the last visit, in those receiving low-moderate intensity LLT with PCSK9i (*n* = 7), monogenic FH had a significantly higher median LDL-C level compared to the polygenic and undetermined groups combined (125 mg/dl vs. 53 mg/dl). In contrast, among individuals receiving high-intensity LLT in combination with PCSK9i (*n* = 30), the monogenic group attained a lower median LDL-C level (50 mg/dl) as compared to the polygenic and undetermined groups (82 mg/dl) [1]. Hence, there was no consistent bias as to which genotype group tended to have a lower or higher therapeutic response upon PCSK9i administration with regard to LDL-C level.

Lee et al. demonstrated a similar result in which the monogenic FH and polygenic hypercholesterolemia responses to alirocumab were not different. In this study, the mean reduction of LDL-C was slightly higher in the polygenic group (3.15 mmol/l (121.8 mg/dl)) compared to monogenic FH (2.94 mmol (113.7 mg/dl)) but not statistically significant (*p* value = 0.62). The percentage reductions in LDL-C in the polygenic and monogenic groups were 67.7% and 63.9%, respectively (*p* = 0.66) [13]. However, the study only involved 39 participants, with a huge discrepancy between the monogenic (*n* = 32) and polygenic (*n* = 7) groups. Thus, this study is arguably underpowered to detect the difference between both groups, and further well-powered studies are necessary. Nevertheless, the result is in support of the previous study [1], indicating that the LDL-C-lowering response to PCSK9i is independent of genotype (monogenic vs. polygenic).

A recent study by Iannuzzo et al. recruited 80 participants with different *LDLR* mutations in a study with either alirocumab or evolocumab. Thirty-nine individuals (48.75%) had defective *LDLR* mutations, while 30 subjects (37.5%) had null *LDLR* mutations, and 11 others (13.75%) were either compound heterozygous or homozygous. The therapeutic response of PCSK9i was evaluated at 2 time points (12 weeks and 36 weeks). At 12 weeks, the percentage of LDL-C level reduction from baseline for defective and null LDLR mutations was − 59.8% and − 60.1%, respectively (*p* value = 1.00). At 36 weeks, the percentage reductions were − 58.2% and − 48.65% for defective and null mutations, respectively (*p* value = 0.54). However, in the compound heterozygous/homozygous group, the LDL-C reduction was less significant relative to defective and null mutations combined, with only − 19.4% and − 23.9% reductions at 12 (*p* value =  < 0.001) and 36 weeks (*p* value = 0.006), respectively [42].

A study involving 1191 patients (898 had identifiable mutations) from 6 clinical trials by Defesche et al. studied the spectrum of genetic mutations of known FH causative genes on alirocumab treatment (758 received alirocumab, 433 were controls). Of all patients with identified mutations, 387 (43%) individuals had defective *LDLR* mutations, 437 (49%) individuals had heterozygous *LDLR* null mutations, 10 (1.1%) subjects had compound *LDLR* mutations, and 1 had a homozygous *LDLR* mutation. Moreover, 46 (5%) patients had *APOB* mutations, 8 (0.9%) patients were with *PCSK9* mutations, two (0.2%) other patients had homozygous mutations in the *LDLR* adaptor protein 1 (*LDLRAP1*) gene, 6 (0.7%) patients were with double heterozygous in the *APOB* and *LDLR* genes, and 1 (0.1%) patient was double heterozygous in the *LDLR* and *PCSK9* genes. After 24 weeks, the LDL-C reductions (in percentage from baseline) were similar across genotypes and in those without identifiable mutations. In the three major genes (*LDLR*, *APOB*, and *PCSK9*), there was no significant difference in the percentage of LDL-C reduction at 12 weeks (*p* value = 0.65), even after adjusting for statin and alirocumab dose [43].

In the ORION-9 trial on inclisiran, genetic testing was carried out on 432 patients, of whom 256 (80.8%) individuals had one *LDLR* causative variant, 23 (5.3%) others had *APOB* variants, and 1 patient had a *PCSK9* gain-of-function variant. Thirty-seven others (8.6%) either were double heterozygous, compound heterozygous, or true homozygous. Of all individuals with single *LDLR* variants, 231 (90.2%) were pathogenic, 17 (6.6%) were probably pathogenic, and 8 (3.1%) were variants of uncertain significance (VUS). The mean values of LDLC reduction between pathogenic, probably pathogenic, and VUS were similar [44].

A summary of available studies on the effect of genotype in the treatment of PCSK9 inhibitors is provided in Table 2.

**Table 2:** The summary of published studies on PCSK9 inhibitor across different genetic backgrounds

Author	Study design	Baseline LLT (*N*)	Genotype classification (*N*)	PCSK9i intervention	Follow-up	Findings
D’Erasmo et al., 2021 [1]	Observational	Low-moderate statins ± ezetimibe (7) High-intensity statins ± ezetimibe (30)	Monogenic FH (FH/M +) (33) Polygenic hypercholesterolemia or genetically undetermined (FH/M-) (4)	Evolocumab or alirocumab	24–34 mo	No significant LDL-C level difference at last visit between FH/M + and FH/M-(*)
Lee et al., 2019 [13]	Observational	Statins (37): (1) No statins (6) (2) Low (1) (3) Moderate (8) (4) High (24) Ezetimibe (26)	Monogenic FH (32) Polygenic hypercholesterolemia (7)	Evolocumab	12 wk	No significant difference in absolute and percent reduction in LDL-C (*)
Iannuzzo el al., 2022 [42]	Observational	Statin (5) Ezetimibe (11) Statin + ezetimibe (50)	He-defective (33) He-null (27) Compound He/HoFH (6)	Evolocumab or alirocumab	36 wk	No significant difference in LDL-C changes in He-defective and He-null subjects Less significant LDL-C reduction in compound He/HoFH, with a significant difference with all the He-FH groups
Defesche et al., 2017 [43]	6 RCTs (Five phase 3 trials are included in the analysis of mutation effect on LDL-C reduction)	Maximally tolerated statins + other LLT in 4 RCTs No background statins in 1 RCT**	*APOB* defective (26) *LDLR* defective (220) *LDLR* negative (256) *PCSK9* GOF (6) Unknown (154)	Alirocumab	24 wk	LDL-C reduction was similar across genotypes and patients without unidentified mutations (*)
Raal et al., 2020/ ORION9 [44]	RCT	Inclisiran (242) (1) Statin (219) (2) High-intensity statin (185) (3) Ezetimibe (135) Placebo (240) (1) Statin (217) (2) High-intensity statin (171) (3) Ezetimibe (120)	Two variants (37) *LDLR* pathogenic (231) *LDLR* probably pathogenic (17) *LDLR* VUS (8) *APOB* variants (23) *PCSK9* GOF variants (1) No variants (115) Untested (50)	Inclisiran	510 days	Those treated with inclisiran, mean LDL-C reduction were similar in all classes of *LDLR* mutations

*GOF*, gain-of-function; *He-defective*, heterozygous defective mutation; *He-null*, heterozygous null mutation; *LLT*, lipid-lowering therapy; *RCT*, randomized controlled trial; *VUS*, variant of uncertain significance.

*No HoFH patients were included. **Study comparing alirocumab vs. ezetimibe on statin-intolerant patients [45]

## Clinical Implications

The current available evidence seems to be in agreement that PCSK9i’s efficacy in reducing LDL-C levels is independent of genetic background. Hence, apart from its favorable and proven efficacy, PCSK9i provides more flexibility to be used in the context of severe hypercholesterolemia, regardless of genotype status (monogenic vs. polygenic vs. unidentified mutation or *LDLR* vs. *APOB* vs. *PCSK9* mutation). This is in contrast with statins as the first-line therapy for hypercholesterolemia, whose degree of therapeutic response is more affected by patients’ genetic background or type of mutation [9–12]. Hence, the genetic diagnosis might be more necessary when PCSK9i therapy is not available, not indicated, or in the initial phase of therapy after establishing a diagnosis to formulate therapeutic options to start with, drug dosing, or LLT intensity based on available genetic information. Afterwards, when PCSK9i is indicated, patients’ genotype is no longer a reliable predictor for LDL-C reduction. Therefore, clinicians should be aware of other variables as predictors of therapeutic response. This was the case in Defesche et al.’s study, in which 92.3% of patients attained ≥ 15% LDL-C reduction at 14 weeks and 96.6% of patients achieved LDL-C reduction of ≥ 15% at least once at 12, 24, and 52 weeks from treatment initiation, regardless of genotype. Among all patients who never achieved ≥ 15% LDL-C reduction, 50% of cases were found to be due to noncompliance and early alirocumab discontinuation before 12 weeks. The majority of those without a clear explanation had identified mutations that were also found in those who managed to achieve ≥ 15% LDL-C reduction in at least one-time point [43]. This supports the notion that genotype is less relevant in predicting the degree of LDL-C reduction on PCSK9i therapy. Moreover, this is also in line with the National Institute for Health and Care Excellence (NICE) guideline, in which non-FH patients are also eligible for PCSK9i therapy when they have known cardiovascular risk factors and persistent hypercholesterolemia (> 4 mmol/l if at high risk for CVD or > 3.5 mmol/l if at very high risk for CVD) [25].

However, this is not without exception. As demonstrated by Iannuzzo et al., a similar response to PCSK9i therapy was observed in heterozygous FH (HeFH) patients, while homozygous FH (HoFH) and compound heterozygous patients demonstrated a lower reduction in LDL-C percentage from baseline [42]. D’Erasmo et al. [1] and Lee et al. [13], who documented similar PCSK9i responses in monogenic FH and polygenic hypercholesterolemia, carried out their studies without any single HoFH patient being included. Hence, these results might only be applicable to HeFH patients. These findings might be exclusively attributed to patients’ genotypes as those with homozygous and compound heterozygous have severely impaired LDLR function and expression [43, 46]. HoFH patients can only have ≤ 2% LDLR function preserved [47]. Defesche et al. also found that > 90% of individuals with compound heterozygous/HoFH receiving alirocumab or evolocumab were also receiving statins and ezetimibe [43]. Therefore, the reduced PCSK9i efficacy in that case was not related to pre-existing background therapy and was most likely due to a severe loss of LDLR function. This is in accordance with a study with evolocumab, concluding that the LDL-C level in HoFH is primarily determined by the residual expression and function of LDLR [48]. Therefore, with HoFH/compound HeFH as an exception, PCSK9i efficacy is likely to be independent of genotype. This notion is, arguably, relevant in the clinical setting as HeFH prevalence is much more common (1:250–500) compared to HoFH (1:1.000.000) [1, 6, 8, 49]. Therefore, in treating HeFH, clinicians need to be aware of other variables as therapeutic success is more dependent on external influences (such as lifestyle, patient compliance, diet, age, or therapeutic dosing) as compared to HoFH, which is heavily dependent on residual LDLR expression and functional reserve [47, 48].

## Is There a Place for PCSK9 Inhibitors for HoFH/Compound Heterozygous FH?

As PCSK9 inhibitors reduce LDLR degradation [19, 50], they require some functional LDLR to work. Consequently, LLTs that increase LDLR upregulation (e.g., statins and PCSK9 inhibitors) tend to have poor lipid-lowering effects in HoFH as the efficacy is highly dependent on residual LDLR function [51]. However, patients’ genotype must not delay therapy, especially in HoFH/compound heterozygous that has the highest atherosclerotic cardiovascular risks from extremely high LDL-C. Statins, with ezetimibe addition, can still be the first-line therapy as they are widely available, more affordable, well-tolerated, might still reduce LDL-C by approximately 25%, and are still capable of improving mortality in HoFH patients [52, 53]. Despite the variability in LDL-C reduction, PCSK9 inhibitors can be given as a second-line therapy after statins [53]. Some studies revealed that around 25% LDL-C reduction might still be attained following PCSK9 inhibitor administration in patients who are already on other background LLTs [29, 54]. Unfortunately, *LDLR* null/null genotype HoFH has virtually no residual LDLR function, hence totally unresponsive to PCSK9 inhibitors [55, 56]. This suggests that generally, PCSK9 inhibitors, with or without statins, might still be beneficial but rarely sufficient for the treatment of HoFH [57], as it requires another therapy that is independent of residual LDLR function. One example is the angioprotein-like 3 (ANGPTL3) inhibitor, evinacumab, that enhances lipoprotein lipase activity to increase VLDL-C degradation. Evinacumab has been shown to reduce LDL-C by almost 50% from baseline [55]. However, as evinacumab is more expensive than PCSK9 inhibitors, PCSK9 inhibitors can be given prior to evinacumab but should be stopped once it is found to be ineffective [53, 56]. However, when cost is not a problem, PCSK9 inhibitor might be skipped, especially when the genotype is null/null HoFH. A new algorithm for the treatment of HoFH has recently been published [56].

## Future Direction

As different genotypes have been shown to have different cardiovascular risk profiles [8], it is necessary to study whether the comparable LDL-C reduction following PCSK9i therapy translates into a similar cardiovascular risk reduction across genotypes. Alirocumab in the ODYSSEY OUTCOME trial involving 18.924 patients (2.8 years of median follow-up) has demonstrated that patients with previous history of acute coronary syndrome (ACS) treated with high-intensity statins demonstrated a significant reduction in the recurrence of ischemic cardiovascular events compared to placebo (9.5% occurrence in alirocumab vs. 11.1% in the placebo group; hazard ratio 0.85; *p* value < 0.001) [27]. Evolocumab was studied in the FOURIER trial, involving 27.564 patients with a documented history of atherosclerotic cardiovascular disease and being evaluated for 2.2 years of median follow-up. The trial demonstrated a significant reduction in cardiovascular risk with evolocumab therapy (with statin as background therapy) as compared to placebo [28]. However, none of these trials stratified the patients based on genotype. Hence, despite evidence suggesting that LDL-C reduction and cardiovascular risk tend to be linear [58], it is still unknown whether the documented cardiovascular risk reduction following PCSK9i treatment will actually be similar or different between genotypes.

Another concern is that all published studies comparing PCSK9i efficacy and cardiovascular risk reduction are either using alirocumab or evolocumab. Judging from disease pathophysiology and inclisiran’s mechanism of action that reduces PCSK9 synthesis to further improve LDLR activity, the efficacy might arguably be similar to that of alirocumab and evolocumab, with exceptions for compound HeFH and HoFH patients. However, further studies on inclisiran are still necessary to prove this notion.

## Conclusion

This review concludes that PCSK9i efficacy in reducing LDL-C levels is, generally, comparable across genotypes, with compound heterozygous and HoFH patients as exceptions. These exceptional findings can be explained by the nature of the genotypes that lead to severe LDLR function loss. Clinically, the comparable PCSK9i efficacy across genotypes makes genotype a less reliable predictor for the degree of LDL-C reduction once PCSK9i is initiated, and attention to other external influences should be paid. However, future work needs to robustly study whether the generally comparable LDL-C-lowering effect across genotypes upon PCSK9i administration leads to similar cardiovascular risk reduction in different genotypes. Also, considering drug availability, cost-effectiveness, safety, tolerability, and genotype, current therapeutic guidelines [56, 59, 60] for hypercholesterolemia that start with statins with additions of other LLTs and are followed by PCSK9 inhibitors are still relevant, including for those with HoFH or compound heterozygous.

## Data Availability

Not applicable for this study.

## Code Availability

Not applicable for this study.
